# Sesamol Protects Testis from Ischemia-Reperfusion Injury through Scavenging Reactive Oxygen Species and Upregulating CREM*τ* Expression

**DOI:** 10.1155/2020/9043806

**Published:** 2020-06-17

**Authors:** Si-Ming Wei, Rong-Yun Wang, Yan-Song Chen

**Affiliations:** ^1^Shulan International Medical College, Zhejiang Shuren University, Hangzhou City, Zhejiang Province 310015, China; ^2^School of Nursing, Zhejiang Chinese Medical University, Hangzhou City, Zhejiang Province 310053, China; ^3^Department of Orthopedics, Zhejiang Xiaoshan Hospital, Hangzhou City, Zhejiang Province 311200, China; ^4^Department of Orthopedics, The Second Affiliated Hospital of Zhejiang Chinese Medical University (Xinhua Hospital of Zhejiang Province), Hangzhou City, Zhejiang Province 310005, China

## Abstract

Testicular torsion/detorsion-induced damage is considered as a typical ischemia-reperfusion injury attributed to excessive reactive oxygen species (ROS) production. ROS may regulate many genes whose expression affects cell-cycle regulation, cell proliferation, and apoptosis. The cAMP-responsive element modulator-*τ* (CREM*τ*) gene expression in the testis is essential for normal germ cell differentiation. The present study was aimed at investigating the effect of sesamol, a powerful antioxidant, on testicular ischemia-reperfusion injury and related mechanisms in an experimental testicular torsion-detorsion rat model. The type of our study was a randomized controlled trial. Sixty rats were randomly divided into the following 3 groups: (1) sham-operated control group (*n* = 20), (2) testicular ischemia-reperfusion group (*n* = 20), and (3) testicular ischemia-reperfusion+sesamol-treated group (*n* = 20). Testicular ischemia-reperfusion was induced by left testicular torsion (720° rotation in a counterclockwise direction) for 2 hours, followed by detorsion. Orchiectomy was performed at 4 hours or 3 months after detorsion. The testis was obtained for the analysis of the following parameters, including malondialdehyde level (a sensitive indicator of ROS), CREM*τ* expression, and spermatogenesis. In the testicular ischemia-reperfusion group, the malondialdehyde level was significantly increased with a concomitant significant decrease in CREM*τ* expression and spermatogenesis in ipsilateral testis. These results suggest that overproduction of ROS after testicular ischemia-reperfusion may downregulate CREM*τ* expression, which causes spermatogenic injury. Sesamol treatment resulted in a significant reduction in the malondialdehyde level and significant increase in CREM*τ* expression and spermatogenesis in ipsilateral testis. These data support the above suggestion. Our study shows that sesamol can attenuate testicular ischemia-reperfusion injury through scavenging ROS and upregulating CREM*τ* expression.

## 1. Introduction

Testicular torsion represents a common urologic emergency. It is seen at any age group but often occurs in male newborns or adolescents between the ages of 13 and 16 years [1]. Testicular torsion is due to a twist of the spermatic cord, which reduces or even completely blocks testicular blood flow. In order to prevent testicular ischemia and necrosis, prompt diagnosis and timely detorsion are necessary to establish blood flow reperfusion of the ischemic testis. In spite of successful detorsion, 12%-68% of cases suffer testicular atrophy and dysfunction [2–4]. Among many mechanisms that explain testicular damage after testicular torsion-detorsion, the most accepted one is ischemia-reperfusion injury [5]. Reactive oxygen species (ROS) overgenerated during ischemia-reperfusion play a crucial role in testicular injury [6, 7]. Excess ROS, including superoxide anion, hydroxyl and peroxyl radicals, and hydrogen peroxide, induce peroxidation of lipids in the cell membrane, protein denaturation, and DNA fragmentation, leading to tissular damage and cellular death [5]. Since testicular germ cells have high content of polyunsaturated fatty acids in plasma membrane, they are vulnerable to ROS damage, especially to lipid peroxidation [8, 9].

To date, some chemicals and drugs, such as trapidil, oxytocin, and modafinil, have been found to be effective in treating testicular ischemia-reperfusion injury in animal models [10–12]. However, none of them have been used clinically to attenuate testicular ischemia-reperfusion injury in patients. Sesamol (3,4-methylenedioxyphenol) is the major component of sesame seed oil [13] and has multiple biological functions, including antioxidant, antiaging, and antidepressant effects [14–18]. It has been used for a number of health problems in ancient Chinese and Indian herbal medicine. It has been demonstrated that sesamol is effective against various diseases, such as myocardial infarction, pulmonary inflammation, cataract, and depression [18–21]. In addition, sesamol, a powerful natural antioxidant, is potently beneficial in treating iron-induced renal and hepatic toxicity and shows no adverse effects [22]. Recent studies reveal that sesamol can protect against cerebral ischemia-reperfusion injury [23, 24]. However, sesamol has never been investigated for its ameliorative effect on testicular ischemia-reperfusion injury. Thus, the present study was aimed at investigating the effect of sesamol on testicular ischemia-reperfusion injury in an experimental testicular torsion-detorsion rat model.

## 2. Materials and Methods

### 2.1. Experimental Animals

Adult male Sprague-Dawley rats (250-300 g; *n* = 60) were purchased from Shanghai SLAC Laboratory Animal Co., Ltd. (Shanghai City, China). They were kept in an air-conditioned room with controlled temperature (21°C ± 1°C), relative humidity (55% ± 5%), and standard light/dark (12/12 h) cycles. All the rats had unrestricted access to standard rodent chow and water. The ethical approval of all the experimental procedures of the study was obtained from our university ethical committee for animal research (Ethical Approval No. 10790). All experiments were done in accordance with international animal ethical rules.

### 2.2. Drugs and Reagents

Sesamol, anti-*β*-actin antibody, ketamine, and hematoxylin and eosin were procured from Sigma Chemical Company (St. Louis, MO, USA). A protein quantification kit was from Bio-Rad Laboratories (Hercules, CA, USA). The anti-cAMP-responsive element modulator-*τ* (CREM*τ*) antibody, horseradish peroxidase-conjugated secondary antibody, and enhanced chemiluminescence detection reagents were supplied by Santa Cruz Biotechnology (Santa Cruz, CA, USA). A malondialdehyde analyzing kit was purchased from Nanjing Jiancheng Bioengineering Institute (Nanjing City, China). Other reagents used in the study were high grade and commercially available.

### 2.3. Experimental Design

A total of 60 Sprague-Dawley rats were randomly divided into the following 3 groups: (1) sham-operated control group (*n* = 20), (2) testicular ischemia-reperfusion group (*n* = 20), and (3) testicular ischemia-reperfusion+sesamol-treated group (*n* = 20). After anesthesia using intraperitoneal injection of ketamine (50 mg/kg), rats were stabilized on the operating table. All experiments were performed under sterile conditions. Through a left-sided ilioinguinal incision, the left testis was delivered out. In the sham-operated control group, an 11-0 atraumatic silk suture was placed through the tunica albuginea. The testis was inserted into the scrotum, and the skin incision was sutured. In the testicular ischemia-reperfusion group, testicular ischemia was induced via twisting the left testis 720 degrees counterclockwise. The testicular ischemic state was maintained by fixing the twisted testis to the scrotum with an 11-0 silk suture. At the end of 2 hours, testicular reperfusion was induced via untwisting the testis to its natural position. The testis was still alive and was placed in the scrotum. The sesamol-treated group received 2-hour testicular ischemia followed by reperfusion and sesamol treatment at the dose of 50 mg/kg through the tail vein at reperfusion. Sesamol was dissolved in 0.05 ml of soybean oil for intravenous injection. The rationale for the chosen dose of sesamol was based on several past studies [19, 21]. At 4 hours following reperfusion, bilateral testes were removed in half of the rats from each group for analysis of the malondialdehyde level, and then the rats were euthanized in the carbon dioxide chamber. At 3 months following reperfusion, testes were collected in the other half of the rats from each group for estimation of cAMP-responsive element modulator-*τ* (CREM*τ*) protein expression and testicular spermatogenesis.

### 2.4. Malondialdehyde Quantification in Testicular Tissue

Testicular tissue was homogenized on ice in a malondialdehyde lysis buffer. The tissue homogenate was centrifuged at 5,000 g for 15 minutes at 4°C, and the supernatant was obtained for the malondialdehyde assay. The protein concentration in the supernatant was analyzed by the Bradford method [25]. To quantify malondialdehyde in testicular tissue, the thiobarbituric acid reactive substance assay was carried out using a commercially available kit according to the manufacturer's instructions [26]. The malondialdehyde concentration was expressed as nmol/mg protein.

### 2.5. Western Blot Assay for CREM*τ* Protein Expression

Testicular tissue was homogenized in ice-cold tissue protein extraction reagents containing 50 mM Tris HCl, pH 7.4, 2 mM sodium orthovanadate, 0.5% sodium deoxycholate, 150 mM NaCl, 1 mM phenylmethylsulfonyl fluoride, 5 *μ*g/ml aprotinin, 1 mM dithiothreitol, 1% nonidet P-40, 0.5 mM ethylenediaminetetraacetic acid, 0.5 *μ*g/ml leupeptin, and 0.1% sodium dodecyl sulfate. After the homogenate was centrifuged at 14,000 g at 4°C for 15 minutes, the supernatant was retained. Protein concentration in the supernatant was detected by using a protein assay kit. The protein sample was denatured by boiling for 3 minutes. Equal amounts of protein samples (20 *μ*g/lane) were resolved by sodium dodecyl sulfate-polyacrylamide gel electrophoresis and then electrotransferred onto a nitrocellulose membrane. After blocking nonspecific binding sites with 5% nonfat dry milk dissolved in Tris-buffered saline containing 0.1% Tween-20 at room temperature for 1 hour, the membrane was immunoblotted overnight at 4°C with primary antibodies targeting CREM*τ* or *β*-actin. The membrane was washed with Tris-buffered saline containing 0.1% Tween-20 and then incubated with horseradish peroxidase-labelled secondary antibody at room temperature for 1 hour. After the membrane was washed again, an enhanced chemiluminescence kit was used to detect protein bands on the membrane. The band intensity was assessed with a GS-700 imaging densitometer (Bio-Rad Laboratories). The intensity ratio of the CREM*τ* band to the internal standard *β*-actin band from the same sample was considered as a relative expression level of the CREM*τ* protein.

### 2.6. Determination of Testicular Spermatogenesis

Testicular weight, seminiferous tubular diameter, number of germ cell layers, and Johnsen's testicular biopsy score were used to determine testicular spermatogenesis. After the testis was removed, it was weighted on a scale. For histopathological evaluation, part of the testis was submersed in Bouin's fixative, dehydrated with the ascending ethanol series, and paraffin-embedded. Then, the 5 *μ*m thick slice was prepared from a paraffin block using a rotary microtome. The slice was stained using hematoxylin-eosin after route deparaffinization and hydration. An investigator, who was blinded to the origin of the testis, evaluated the testicular section using a light microscope. The 20 seminiferous tubules with round or nearly round cross-sections in each section were selected and appraised by measuring the seminiferous tubular diameter, number of germ cell layers, and Johnsen's testicular biopsy score. The seminiferous tubular diameter was estimated using a microscope with an ocular micrometer. The number of germ cell layers in each seminiferous tubule was measured by counting the germ cell layer numbers from the basal membrane to the tubular lumen at 90°, 180°, 270°, and 360° and calculating the mean number. The histological findings in the seminiferous tubule were graded according to Johnsen's testicular biopsy scoring system based on the maturity of the germinal epithelium [27]. Each seminiferous tubule was given Johnsen's score ranging from 1 to 10 [27]. Complete absence of cells in the seminiferous tubule is given a score of 1. Full spermatogenesis with a lot of spermatozoa, germinal epithelium of a regular thickness, and open tubular lumen is given a score of 10.

### 2.7. Statistical Analysis

Results of the experiments were presented as mean ± standard deviation. The GraphPad Prism version 4.0 software (GraphPad Software Inc., San Diego, CA, USA) was used for statistical analysis. One-way analysis of variance was used to compare data among groups, and Student-Newman-Keuls multiple comparison test was used as a post hoc test. Bartlett's test for equal variances was used to find that data distribution of each group was normal. Student *t*-test was used to determine the differences between ipsilateral and contralateral testes within the group. The differences were considered statistically significant at a *P* value of less than 0.05.

## 3. Results

### 3.1. Malondialdehyde Level in Testicular Tissue

**Figure 1:**
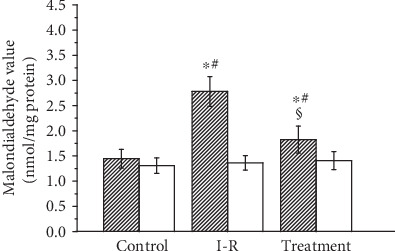
Malondialdehyde analysis of ipsilateral (hatched histograms) and contralateral (open histograms) testes in sham-operated control, testicular ischemia-reperfusion (I-R), and sesamol-treated groups. Values (*n* = 10) are presented as mean ± standard deviation. ∗ means *P* < 0.05 as compared with the control group. # means *P* < 0.05 as compared with contralateral testes in the same group. § means *P* < 0.05 as compared with ipsilateral testes in the I-R group.

The malondialdehyde level in testicular tissue in the sham-operated control, testicular ischemia-reperfusion, and sesamol-treated groups is summarized in Figure 1. Compared with the sham-operated control group, the malondialdehyde level in ipsilateral testes was found to be significantly higher in the testicular ischemia-reperfusion group (*P* < 0.05). Sesamol treatment significantly decreased the malondialdehyde level in ipsilateral testes as compared with the testicular ischemia-reperfusion group (*P* < 0.05). The malondialdehyde level in contralateral testes did not differ significantly among three groups (*P* > 0.05).

### 3.2. Testicular CREM*τ* Protein Expression

**Figure 2:**
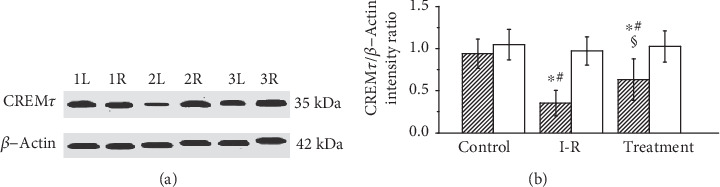
Analysis of CREM*τ* protein expression in ipsilateral (hatched histograms) and contralateral (open histograms) testes in sham-operated control, testicular ischemia-reperfusion (I-R), and sesamol-treated groups. (a) Representative Western blots of CREM*τ* protein expression in rat testes, where *β*-actin is used as loading control. Lanes 1L and 1R: left (i.e., ipsilateral) and right (i.e., contralateral) testes in sham-operated control group; Lanes 2L and 2R: ipsilateral and contralateral testes in testicular I-R group; Lanes 3L and 3R: ipsilateral and contralateral testes in sesamol-treated group. (b) The intensity ratio of CREM*τ* band to *β*-actin band from the same sample is considered as a relative expression level of CREM*τ* protein. Values (*n* = 10) are presented as mean ± standard deviation. ∗ means *P* < 0.05 as compared with the control group. # means *P* < 0.05 as compared with contralateral testes in the same group. § means *P* < 0.05 as compared with ipsilateral testes in the I-R group.

As shown in Figure 2, CREM*τ* protein expression in ipsilateral testes significantly decreased in the testicular ischemia-reperfusion group as compared with the sham-operated control group (*P* < 0.05). Sesamol treatment significantly improved CREM*τ* protein expression in ipsilateral testes as compared with the testicular ischemia-reperfusion group (*P* < 0.05). The CREM*τ* protein expression in contralateral testes did not differ significantly among the three groups (*P* > 0.05).

### 3.3. Findings of Testicular Spermatogenesis

**Figure 3:**
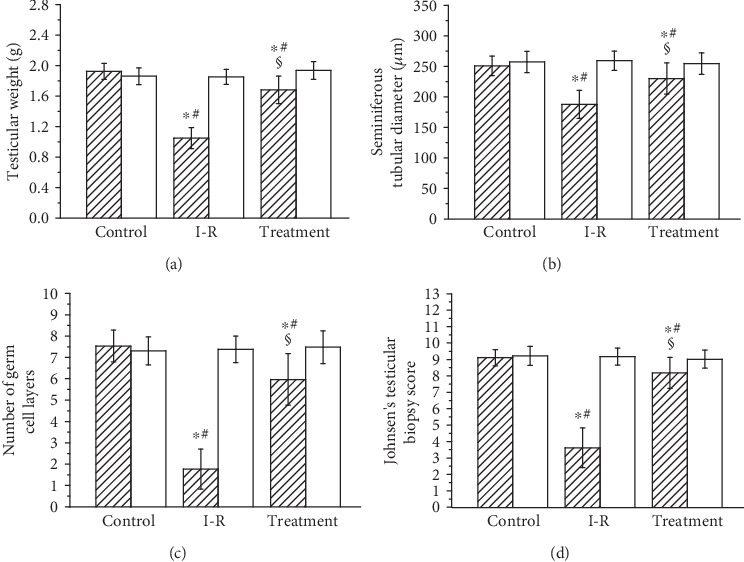
Analysis of testicular weight (a), seminiferous tubular diameter (b), number of germ cell layers (c), and Johnsen's testicular biopsy score (d) in ipsilateral (hatched histograms) and contralateral (open histograms) testes in sham-operated control, testicular ischemia-reperfusion (I-R), and sesamol-treated groups. Values (*n* = 10) are presented as mean ± standard deviation. ∗ means *P* < 0.05 as compared with the control group. # means *P* < 0.05 as compared with contralateral testes in the same group. § means *P* < 0.05 as compared with ipsilateral testes in the I-R group.

**Figure 4:**
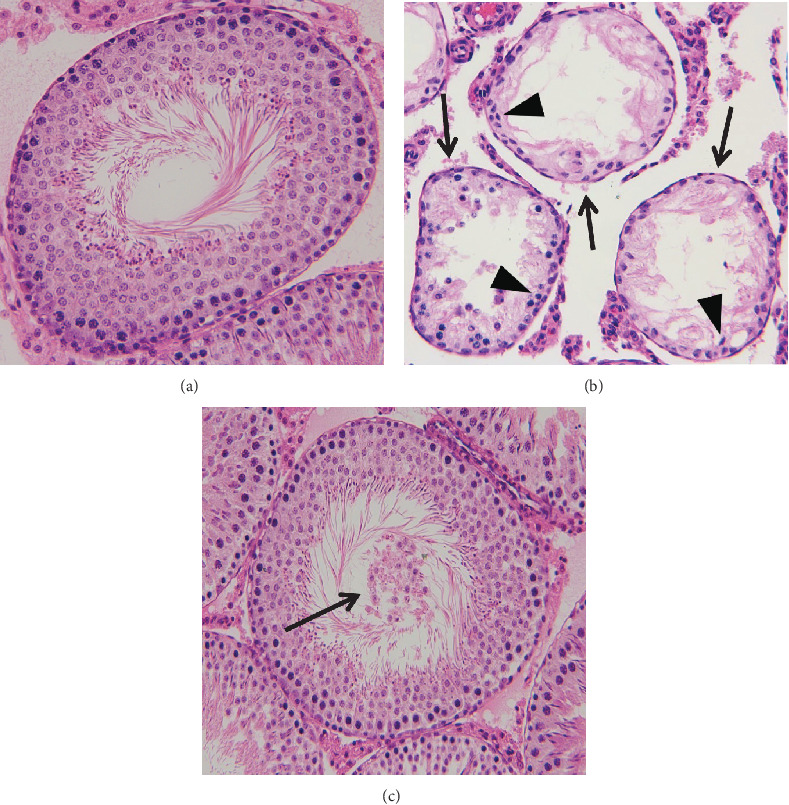
Histopathological sections of testicular tissue in sham-operated control, testicular ischemia-reperfusion, and sesamol-treated groups. Cross-sections of testicular tissue were stained using hematoxylin-eosin and evaluated microscopically at magnification ×200. (a) The histopathological examination revealed normal seminiferous tubular diameter, number of germ cell layers, and spermatogenesis from immature germ cells to mature spermatozoa in bilateral testes of the control group and contralateral testes of testicular ischemia-reperfusion and sesamol-treated groups. An open tubular lumen was observed at the center of the seminiferous tubule. (b) Abnormal testicular morphology with an atrophic seminiferous tubule (arrows), decreased number of germ cell layers (arrowheads), and maturation arrest of germ cells (arrowheads) was seen in ipsilateral testes of the testicular ischemia-reperfusion group. (c) The general appearance of ipsilateral testes in the sesamol-treated group was similar to the control group. However, sloughed germinal cells were present in the tubular lumen (arrow), which led to obstruction of the tubular lumen easily.

In testicular ischemia-reperfusion groups, significant decrease in testicular weight, seminiferous tubular diameter, number of germ cell layers, and Johnsen's testicular biopsy score in ipsilateral testes were seen as compared with the sham-operated control group (*P* < 0.05, Figures 3 and 4). Sesamol treatment significantly improved the four parameters in ipsilateral testes when compared with the testicular ischemia-reperfusion group (*P* < 0.05). The four parameters in contralateral testes did not differ significantly among three groups (*P* > 0.05).

## 4. Discussion

The incidence rate of testicular torsion is 1 in 4000 males under 25 years old [28]. Early diagnosis and urgent surgical detorsion are crucial for the preservation of testicular reproductive function. If testicular torsion persists for longer than 6 hours, it will lead to testicular infarction [29]. Testicular atrophy will develop in 27% of the patients even if surgical detorsion is performed within 5 hours after the appearance of symptoms [4]. In our study, the ipsilateral testis appeared to be viable after 2 hours of unilateral testicular torsion followed by detorsion. However, unilateral testicular torsion-detorsion induced severe spermatogenic injury in the ipsilateral testis 3 months after detorsion, which was characterized by significant reduction in testicular weight, seminiferous tubular diameter, number of germ cell layers, and Johnsen's testicular biopsy score.

Testicular torsion-detorsion is considered as a typical ischemia-reperfusion process for the testis. Testicular ischemia-reperfusion brings about overproduction of ROS [6, 7]. Excessive amounts of ROS may oxidize DNA, membrane lipids, and proteins, which can induce cellular damage [5]. ROS are extremely difficult to quantify directly because of their high reactivity and short life span [30]. Malondialdehyde, which is a stable end product of lipid peroxidation generated by ROS, is widely accepted as a sensitive indicator of ROS [31, 32]. In the present study, we found a significant rise in the testicular malondialdehyde level and a significant reduction in spermatogenesis in the ipsilateral testis following unilateral testicular ischemia-reperfusion. These findings suggest that excessive ROS production is responsible for testicular ischemia-reperfusion injury. ROS scavenger may provide protection against ischemia-reperfusion injury [33, 34].

Sesamol is a powerful antioxidant with high scavenging activity for ROS [35–40]. The antioxidant property of sesamol is due to the presence of phenolic groups in its chemical structure, which can scavenge the superoxide anion [35–37]. In addition, sesamol possesses a benzodioxyl moiety in the structure, which is responsible for scavenging hydroxyl and peroxyl radicals [35, 36, 38–40]. Recent studies have demonstrated a beneficial effect of sesamol on cerebral ischemia-reperfusion injury [23, 24]. Consequently, we made an attempt to investigate whether sesamol has a protective effect against testicular ischemia-reperfusion injury. In our study, sesamol administration decreased the malondialdehyde level and increased spermatogenesis in the ipsilateral testis. These findings show that sesamol plays a protective role in testicular ischemia-reperfusion injury via its antioxidant activity.

The CREM gene encodes various CREM proteins by alternative exon splicing and alternative translational initiation [41, 42]. The full-length CREM protein is named CREM*τ* which contains two glutamine-rich transcriptional activation domains and acts as a transcriptional activator [43]. The truncated CREM proteins lacking glutamine-rich transcriptional activation domains function as transcriptional antagonists [43]. The transcriptional activator CREM*τ* is highly expressed in the adult testis, while in the prepubertal testis, only the antagonist forms of CREM are detected at low levels [43]. The CREM*τ* protein relies on its glutamine-rich domains to mediate transcriptional activation of several germ cell-specific genes required for the structuring of the spermatozoon, such as protamine, transition protein, mitochondrial capsule selenoprotein, calspermin, and RT7 [43–45]. Hence, CREM*τ* acts as a master switch governing male germ cell differentiation [46]. To clarify the crucial function of CREM*τ* in spermatogenesis, mutant mice lacking CREM*τ* expression in the testis were generated by homologous recombination [44]. Testes from CREM*τ*-deficient mice show a reduction of 20%-25% in weight [44]. Analysis of the seminal fluid from CREM*τ*-deficient mice demonstrates absence of mature spermatozoa [44]. Seminiferous tubules from CREM*τ*-deficient mice display a decrease of 20%-30% in diameter [44]. Analysis of the seminiferous epithelium in CREM*τ*-deficient mice reveals that spermatogenesis stops at the early stage [44]. Thus, CREM*τ* expression in the testis is necessary for a normal spermatogenesis [44]. Our study found that ipsilateral testicular spermatogenesis significantly decreased 3 months after unilateral testicular ischemia-reperfusion, including significant reduction in testicular weight, seminiferous tubular diameter, number of germ cell layers, and Johnsen's testicular biopsy score. Moreover, the ipsilateral testis with spermatogenic injury also showed reduced CREM*τ* expression. Because CREM*τ* expression in the testis is necessary for a normal spermatogenesis as described in CREM*τ*-deficient mice [44], a decrease in ipsilateral testicular CREM*τ* expression after unilateral testicular ischemia-reperfusion may result in testicular spermatogenic injury. However, the reason for decrease in CREM*τ* expression still remains unknown.

Excess ROS are generated after testicular ischemia-reperfusion [6, 7]. ROS may regulate many genes whose expression affects cell-cycle regulation, cell proliferation, and apoptosis [47]. The CREM*τ* gene expression affects testicular spermatogenesis [44], and a decrease in ipsilateral testicular CREM*τ* expression after unilateral testicular ischemia-reperfusion may result in testicular spermatogenic injury, as discussed above. When all these results were taken together, we hypothesized that overgeneration of ROS after testicular ischemia-reperfusion might downregulate CREM*τ* expression, resulting in spermatogenic injury. In the present study, we found that treatment with sesamol, which is a potent ROS scavenger, significantly decreased the malondialdehyde level (a sensitive indicator of ROS) and significantly enhanced CREM*τ* expression and spermatogenesis in the ipsilateral testis, compared with those in the testicular ischemia-reperfusion group. These data confirm the above hypothesis. At the same time, these data also suggest that sesamol has a protective effect against testicular ischemia-reperfusion injury by upregulating CREM*τ* expression by scavenging ROS.

Whether unilateral testicular ischemia-reperfusion affects the contralateral testis is still controversial. Although some investigators have demonstrated that the contralateral testis is damaged after unilateral testicular ischemia-reperfusion [48, 49], other investigators have not observed this phenomenon [50, 51]. In the current study, we found significant changes in the malondialdehyde level, CREM*τ* expression, and spermatogenesis in ipsilateral testicular tissue after unilateral testicular ischemia-reperfusion. Nevertheless, we observed no marked changes in these parameters in the contralateral testis. These results indicate that unilateral testicular ischemia-reperfusion has no detrimental effect on the contralateral testis.

Some investigators added a vehicle group lacking the sesamol in their study of sesamol [52], whereas other investigators did not [53]. The vehicle did not show any effect in their study [52]. We used soybean oil as the vehicle, and sesamol was dissolved in 0.05 ml of soybean oil for intravenous injection. We searched articles about soybean oil before our study started. None of these articles showed that soybean oil had effects on rat testicular spermatogenesis and testicular ischemia-reperfusion injury. Therefore, we chose soybean oil as the vehicle in the study. Also, for this above-mentioned reason, we did not add a vehicle group without the sesamol in the study. However, adding a vehicle group in the study is the best research design. We will add a vehicle group in a future study.

Soybean oil-based intravenous lipid emulsion plays key clinical roles in parenteral nutrition treatment not only as a source of nonprotein calories but also as a preventive therapy for essential fatty acid deficiency [54]. Inoue et al. have reported that tail intravenous injection of soybean oil in 0.05 ml volume does not cause pulmonary fat embolization and lung injury in rats [55]. In our study, sesamol was dissolved in 0.05 ml of soybean oil for tail intravenous injection in rats. We did not find rat death after intravenous injection of soybean oil. Hence, intravenous injection of soybean oil in 0.05 ml volume is safe for rats.

A group receiving sesamol alone can help investigators to find the basal effect of sesamol on the normal testis. In our unilateral testicular ischemia-reperfusion+sesamol-treated group, we found that sesamol treatment had a protective effect on the ipsilateral ischemia-reperfusion testis. However, sesamol had no effect on the contralateral normal testis. Therefore, this group receiving sesamol alone may be left out in the present study.

Some studies showed that using ketamine alone as an anesthetic was effective in making a rat model of testicular ischemia-reperfusion injury [56–58]. Consequently, we also used ketamine alone as the anesthetic during rat testicular operation, and our surgical operation also verified its effectiveness.

It has been reported that renal ischemia-reperfusion increases beta-actin expression [59]. However, many studies about testicular ischemia-reperfusion showed that testicular ischemia-reperfusion had no effect on beta-actin expression [7, 60–63]. As a result, many investigators used beta-actin as an internal control in the study of testicular ischemia-reperfusion [7, 60–63]. Because the testis and kidney are different organs, ischemia-reperfusion of the testis and kidney has different effects on beta-actin expression. Based on the above-mentioned reason, we used beta-actin as the internal control in our study of testicular ischemia-reperfusion.

Under normal conditions, the cellular proteins are kept separate from proteases, which are naturally present in cells. However, cellular architecture is destroyed during protein extraction. The endogenous proteases are released from subcellular compartments and degrade cellular proteins. Degradation of proteins can reduce protein yield and affect protein assay. To ensure that the best protein yield is obtained, protease inhibitors are used for protection of proteins against protease degradation during protein extraction. Therefore, protease inhibitors do not affect the protein assay. On the contrary, they are beneficial to the protein assay. In our study, we used many protease inhibitors, such as aprotinin, leupeptin, and phenylmethylsulfonyl fluoride. These protease inhibitors protect proteins against a wide range of proteases.

Each seminiferous tubule was given Johnsen's score ranging from 1 to 10 [27]. Complete absence of cells in the seminiferous tubule is given a score of 1. Full spermatogenesis with a lot of spermatozoa, germinal epithelium of a regular thickness, and open tubular lumen is given a score of 10. Therefore, Johnsen's testicular biopsy scores are quantitative data instead of qualitative data. We can use quantitative tests such as the one-way analysis of variance to test them.

We used Bartlett's test for equal variances to find that data distribution of each group was normal. In some articles, Bartlett's test for equal variances was also used to find that data distribution of each group was normal [64–66]. These articles support our test.

In conclusion, our study demonstrates that overgeneration of ROS after testicular ischemia-reperfusion can do harm to testicular spermatogenesis by downregulating CREM*τ* expression. Furthermore, we reveal that sesamol protects testis from ischemia-reperfusion injury through scavenging ROS and upregulating CREM*τ* expression. We hope that sesamol can become the first pharmacological agent in clinical practice to treat patients suffering from testicular ischemia-reperfusion injury. However, further clinical studies are necessary to verify the therapeutic potential of sesamol.

## Data Availability

The data used to support the findings of this study are included within the article.
